# Continuity of Visiting Pharmacist Services for Older Adults With and Without Terminal Cancer in Japan

**DOI:** 10.1093/geroni/igaf122.2687

**Published:** 2025-12-31

**Authors:** Reina Taguchi, Akira Okada, Rumiko Tsuchiya-Ito, Satomi Kitamura, Tomoki Ishikawa, Shota Hamada

**Affiliations:** Institute for Health Economics and Policy, Association for Health Economics Research and Social Insurance and Welfare, Tokyo, Tokyo, Japan; The University of Tokyo, Tokyo, Tokyo, Japan; Dia Foundation for Research on Ageing Societies, Tokyo, Tokyo, Japan; Institute for Health Economics and Policy, Association for Health Economics Research and Social Insurance and Welfare, Tokyo, Tokyo, Japan; Institute for Health Economics and Policy, Association for Health Economics Research and Social Insurance and Welfare, Tokyo, Tokyo, Japan; Institute for Health Economics and Policy, Association for Health Economics Research and Social Insurance and Welfare, Tokyo, Tokyo, Japan

## Abstract

Homebound older adults often experience polypharmacy and complex medication regimens. Visiting pharmacist services, which support in-home medication management, have been reported to help optimize medication use, but little is known about their continuity. This study aimed to examine the continuity of visiting pharmacist service and reasons for cessation in a homebound older population in Japan stratified by terminal cancer status. This retrospective cohort study used medical and long-term care claims data from older adults aged ≥65 years in Hachioji-city of Tokyo, Japan, who began to receive visiting pharmacist services between April 2014 and March 2019. Participants were followed until service cessation (absence of service claims for two consecutive months) or 12 months from initiation. Reasons for cessation were categorized into death, relocation, hospitalization/institutionalization, or discontinuation. The study included 3,952 participants, with 353 (8.9%) having terminal cancer. The median time to any-cause cessation was 2 months for participants with terminal cancer, while 51.9% of those without terminal cancer remained event-free at study end. Death was the most common reason, but the 12-month cumulative incidence for participants with terminal cancer was more than four times higher. For individuals with terminal cancer, the service typically begins months before death, requiring pharmacists to adapt to condition changes. Those without cancer often use the service for over a year, necessitating routine medication review. These findings will help pharmacists tailor care to individual patient needs and expected service duration. They can also guide countries considering similar services in response to aging populations and healthcare shortages.

